# Considering Future Potential Regarding Structural Diversity in Selection of Forest Reserves

**DOI:** 10.1371/journal.pone.0148960

**Published:** 2016-02-11

**Authors:** Johanna Lundström, Karin Öhman, Mikael Rönnqvist, Lena Gustafsson

**Affiliations:** 1 Department of Forest Resource Management, Swedish University of Agricultural Sciences, Umeå, Sweden; 2 Département de génie mécanique, Université Laval, Québec, Canada; 3 Department of Ecology, Swedish University of Agricultural Sciences, Box 7044, SE-750 07, Uppsala, Sweden; Chinese Academy of Forestry, CHINA

## Abstract

A rich structural diversity in forests promotes biodiversity. Forests are dynamic and therefore it is crucial to consider future structural potential when selecting reserves, to make robust conservation decisions. We analyzed forests in boreal Sweden based on 17,599 National Forest Inventory (NFI) plots with the main aim to understand how effectiveness of reserves depends on the time dimension in the selection process, specifically by considering future structural diversity. In the study both the economic value and future values of 15 structural variables were simulated during a 100 year period. To get a net present structural value (NPSV), a single value covering both current and future values, we used four discounting alternatives: (1) only considering present values, (2) giving equal importance to values in each of the 100 years within the planning horizon, (3) applying an annual discount rate considering the risk that values could be lost, and (4) only considering the values in year 100. The four alternatives were evaluated in a reserve selection model under budget-constrained and area-constrained selections. When selecting young forests higher structural richness could be reached at a quarter of the cost over almost twice the area in a budget-constrained selection compared to an area-constrained selection. Our results point to the importance of considering future structural diversity in the selection of forest reserves and not as is done currently to base the selection on existing values. Targeting future values increases structural diversity and implies a relatively lower cost. Further, our results show that a re-orientation from old to young forests would imply savings while offering a more extensive reserve network with high structural qualities in the future. However, caution must be raised against a drastic reorientation of the current old-forest strategy since remnants of ancient forests will need to be prioritized due to their role for disturbance-sensitive species.

## Introduction

There is a widely recognized need to protect structurally diverse forests since they may host a rich biodiversity [1]. Such forests are declining globally due to fragmentation and reduction of natural habitats caused by anthropogenic disturbances [2]. An important part of conservation is to establish reserves, which ideally should be persistent and representative of natural habitats in their respective areas [3]. However, the requirements for reserves, in terms of areas needed to protect all threatened habitats, is large. For instance the Aichi target of Convention on Biological Diversity states that at least 17% of the terrestrial land area should be protected until year 2020 [4] implying large economic costs to establish and maintain them. Thus, reserves need to be planned as effectively as possible to maximize fulfillment of conservation targets. Use of a systematic approach can increase the effectiveness of these procedures [3], and there have been rapid developments, theoretical and methodological, concerning ways to improve reserve selection during the last 20 years [5,6].

Most Northern European boreal forests are intensively managed, and have been so for a long time, leading to even-aged forests with small amounts of features that are common in natural forests [7–9]. A forest reserve network should ideally represent the natural range of forest structures and composition as well as successional stages in its geographic area [10,11]. Conservation qualities will change over time within the selected reserves. A time discounting strategy is therefore needed to enable evaluation of both present and future effects of different conservation strategies on states and properties of the reserves. Further, if the conservation qualities are believed to be degenerating in the landscape and assuming a decreasing marginal utility of the qualities, they will be more valuable to protect for future generations than the present (in contrary to the wealth effect justifying economical discounting [12]), motivating a smaller discount rate for environmental change than consumption change [13]. Key factors to inform conservation decisions include differences in the economic value of the land [14], and the variability of conservation targets [15]. Environmental variables can serve as surrogates for biodiversity [16–18] for forests represented by factors like dead wood and old trees [19–21]. Climate change further complicates effective conservation planning, as it could alter habitat conditions sufficiently seriously to drive species from protected areas [22,23].

In a previous paper, we identified the most cost-effective age composition of reserves in boreal Sweden based on a reserve selection model and a set of structural indicators [24]. The results indicated that it would be cost-effective to select a large proportion of young forests, since they host all indicators and are cheaper to purchase than older forests, where the indicators are denser but the price is much higher. However, the model did not consider the future development of the indicators in the candidate reserves.

The aim of the study presented here was to investigate the effects of considering future habitat potential, expressed as structural richness, on the effectiveness of reserves by adding a time dimension to the selection process [24]. We recalculated future structural values back in time, (i.e. discounted values), to handle uncertainties [25,26], by adapting the net present biodiversity value (NPBV) [27] to net present structural value (NPSV). The current practice of forest reserve selection is almost exclusively based on current forest structure and composition. Thus, our study contributes to the development of a selection strategy based on forest protection goals set for future generations. Our hypotheses were as follows. First, conservation effectiveness (estimated as accumulated structural indicator score over 100 years) will be higher if future values are heavily weighted in the NPSV. Second, young forests are more cost-effective reserve options if their future structural value is taken into account. The rationale for this hypothesis is that young forests are cheaper than older forests so a larger total area can be purchased, increasing the potential growth of indicators, and some indicators will increase more rapidly in young than in old forests.

## Materials and Methods

### Study area

Our study was confined to boreal Sweden [28], which covers approximately 14 million ha of productive forest land. Boreal forests, both natural and managed, have low tree species diversity and are dominated by the conifers Scots pine *Pinus sylvestris* L and Norway spruce *Picea abies* (L) Karst. [29]. The main deciduous trees are silver birch *Betula pendula* Roth., downy birch *B*. *pubescens* Ehrh., and aspen *Populus tremula* L. [30].

We used forest data from 17,599 temporary and permanent Swedish National Forest Inventory (NFI) plots on all types of productive forest land, outside existing reserves and inventoried in 2003–2007 (S1 Dataset). The NFI is an annual survey, which started in 1923, of circular plots with a radius of 7 or 10 m placed along borders of tracts distributed in a systematic cluster design across all land in Sweden [31].

We aggregated the NFI plots in the Boreal region into 112 larger 50x50 km squares for the analyses to get a more realistic representation of the forest, since a small plot can have extreme values. Each aggregated 50x50 km square had to contain at least 30 NFI plots, leading to the exclusion of 292 NFI plots at the edges of the study area. We divided the forest within each aggregated 50x50 km square into five age classes: 0–14, 15–39, 40–69, 70–99 and ≥100 years. We selected the 100-year timeframe and unequal class ranges because the normal rotation time in the region is about 100 years and large-scale tree retention practices, i.e. retention of living and dead trees during forestry operations for conservation purposes, [32] were introduced approximately 15 years before the study [33]. Older managed forests have much less retained structures [34], and thus different initial characteristics.

We considered all NFI variables and identified 15 of these as structural variables that reflect qualities that are important for a substantial proportion of the forest species [17,35,36]. The value of each indicator was translated into a point between 0 and 100 (Table 1) to enable calculations. Each aggregated 50x50 km square was assigned a point per hectare for each of the 15 variables and age-class based on the points from the including NFI-plots. We assumed that maximizing the points for the 15 indicators would increase the potential for high species richness, including rare species.

**Table 1:** List of forest structural indicators, criteria for points, and the discount rate used in the discount function 3 (see Material and Methods).

Indicator	100 points	50 points	0 points	Discount rate (%)
Uneven age^a^	Uneven-aged		Even-aged	0.1
Tree layer^b^	Gini coefficient > 0.5	Gini coefficient ≤ 0.5^c^		0.1
Ground structure^d^*	Very uneven/fairly uneven	Fairly even	Very even	0.1
Large pine	>40 cm dbh	>30 cm dbh	Not present	1
Large spruce	>40 cm dbh	>30 cm dbh	Not present	2
Large birch	>40 cm dbh	>30 cm dbh	Not present	0.5
Large aspen	>40 cm dbh	>30 cm dbh	Not present	0.5
Large deciduous tree (other than birch or aspen)	>40 cm dbh	>30 cm dbh	Not present	0.5
Dead conifer tree lying	1 m^3^/ha dead trees >20 cm dbh^e^		Not present	0.5
Dead deciduous tree lying	1 m^3^/ha dead trees >20 cm dbh^e^		Not present	0.5
Dead conifer tree standing	1 m^3^/ha dead trees >20 cm dbh^e^		Not present	1
Dead deciduous tree standing	1 m^3^/ha dead trees >20 cm dbh^e^		Not present	1
Presence of rowan*	Present		Not present	0.1
Affected by water (moving water/spring/temporarily flooded)*	Yes		No	0.1
Volume of dead wood^f^	>20 m^3^/ha	≤ 20 m^3^/ha^c^		0.5

a If at least 80% of the volume is within a 20-year age range the stand is even aged, otherwise uneven-aged.

b Based on the Gini coefficient, which is a measure of heterogeneity and can be used to describe size hierarchies and to quantify the deviation from perfect equality. The Gini coefficient ranges from 0 to 1, where 0 is perfect equality and 1 is maximal inequality

c Normalized points from 0–100.

d Ground structure: Classification based on height and frequency of landscape feature irregularities (rocks, hillrocks and holes) on the ground.

e 1 m3/ha corresponds to approximately one tree per ha, and the dbh limit is based on data in Sweden’s statistical yearbook of forestry [54].

fThe indicator “volume of dead wood” was assigned 100 points when the volume was >20 m3/ha in a 50x50 km square, and normalized points ranging from 0–100 when the volume was lower. This difference in point setting was motivated by indications that 20 m3/ha could be a threshold level for many species associated with dead wood [55,56].

*Variables only registered in period 0 and not projected.

### Stand development simulations

The future development of the 15 structural indicators in each NFI plot was simulated for 100 years using the Heureka system which is a decision support system developed for planning and analysis of multiple-use forestry [37]. Heureka is based on projections of the tree cover development. To predict the future state of the forest, data on current conditions, applied management actions (e.g. thinning, clear-cutting and appropriate regeneration measures following harvest), and known ecological processes (e.g. tree growth, mortality and climate change) are used. For a more detailed description see Wikström et al. [37]. The 15 indicators’ future estimated values were projected in 5-year periods for 100 years. The value of each indicator was translated into the corresponding points (Table 1) for each 5-year period. In this paper we use value when referring to the measured value in an NFI plot, points when referring to an assigned value, and score when referring to a sum of points. We aggregated the points for the 50x50 km squares divided into the five forest age classes, i.e. each aggregated 50x50 km square was assigned points per ha for each indicator, forest age class and period.

The opportunity cost of each age class and aggregated 50x50 km square was also estimated per ha based on the cost of setting aside the including NFI plots. The cost of setting aside the NFI plots was based on the estimated economic value of the most profitable forestry connected to the plot. For each NFI plot we simulated up to 50 different treatment schedules (i.e. sequences of future treatments, such as thinning, clear-cutting or doing nothing) with the Heureka system. For each schedule the net present value (NPV), i.e. the predicted income from future activities in the plot minus the predicted cost of future activities from period 1 to infinity, discounted back to today with a 3% interest rate, was calculated. The schedule yielding the highest NPV was then chosen to represent the opportunity cost for that plot. The cost and income were based on Heureka’s default timber price list for northern Sweden, and if only negative NPVs were obtained for a plot we set its opportunity cost to 0, since it would not be realistic to have negative values in the model.

### Net Present Structural Value

We adapted the net present biodiversity value concept NPBV [27] to estimate a single structural value NPSV that included the values from future time periods for each indicator. This was done by applying a time discount to future values.

We calculated NPSVs for each indicator (*e*) as follows:

$${NPSV}_{e}=p_{n}\cdot f\left(n\right)$$ (1)

Where *p*_*n*_ is the projected value *n* years from now and the function *f(n)* is the discount function.

We used four versions of the discount function. Discount function 1): (*f*_*1*_) (Eq 2) only today´s value was used, i.e. only the first period (*j*_*0*_) was considered, ignoring future values and risks of future losses:

$$f_{1}\left(n\right)=j_{n}\;j_{0}=1,\;j_{n}=0,\;n=1,\ldots \;,100$$ (2)

Discount function 2): (*f*_*2*_) (Eq 3) the same weight was applied to every time period, i.e. future values were considered, but not risk:

$$f_{2}\left(n\right)=1\;n=0,\ldots \;,100$$ (3)

Discount function 3): (*f*_*3*_) (Eq 4) risk was considered by applying a discount rate. Discount rate is an economic concept that can be interpreted as the fraction of the value of a resource that is lost when choosing to consume it in the future instead of today, in a similar manner to our use of an interest rate in the net present value calculations to establish the opportunity cost. Each indicator was assigned a discount rate based on the likelihood of it being negatively affected by a storm (Table 1); the major kind of disturbance in boreal Sweden today [30]. Deciding appropriate discount rates is inherently problematic because of the uncertainties regarding the magnitude (or even nature) of possibly influential variables, and discounting environmental variables is a new idea for which no consensus regarding suitable methodologies has been reached yet [27,13,38]. However, we assigned discount rates of 2%, 1% and 0.5% to spruce, pine and deciduous tree species, respectively. The discount rates were based on findings by Peltola et al. [39] regarding the relative sensitivity of tree species to storms and indications that discount rates for ecological changes probably is lower than the generally used economical interest rate 3% [13,27]. We assumed that standing dead trees would be more sensitive than fallen trees, and the remaining indicators were assigned a small discount rate of 0.1% to account for the risk of indirect effects. There are indications that tree damage caused by storms will increase in the future due to climate change [40], thus the discount rate for all indicators was increased linearly by 1% over 100 years (i.e. by 0.01% for each year). In this version of the discount function a discount rate (*d*) that increases with time was applied, i.e. we added an additional risk rate that differed for each indicator (*e*) (Table 1), but for simplicity we still describe the function as:

$$f_{3}\left(n\right)={\left(1-\left(d_{e}+\frac{0.01\times n}{100}\right)\right)}^{n}$$ (4)

Discount function 4): (*f*_*4*_) (Eq 5) only the projected value for year 100 was used, i.e. neither risk nor any present or future values until year 100 where considered:

$$f_{4}\left(n\right)=j_{n}\;j_{100}=1,\;j_{n}=0,\;n=0,\ldots \;,99$$ (5)

### Reserve-selection model

The approach for selecting nature reserves was based on the model presented in Lundström et al. [24], including structural indicators (Table 1). However, here the original model is complemented with a time dimension and the option to add a discount function. The model is based on a goal programming approach, where the objective is to select areas that maximize structural potential, considering all indicators simultaneously. The solution to the problem is divided into two phases: in the first a maximal value for all indicators is established, and in the second a solution is found that minimizes the sum of the deviations from each indicator’s maximal value and the final value. The linear programming (LP) problem used in phase one [P1] can be formulated as follows (see Table 2 for parameters and decision variables):

$$\lfloor P1\rfloor \;\text{max}\;z=\sum_{e\in E}\sum_{j\in J}\sum_{i\in I}\sum_{t\in T}w_{e}p_{jit}x_{it}f_{ej}$$ (6)

Subject to the following constraints:

$$\sum_{i\in I}\sum_{t\in T}c_{it}x_{it}\leq b$$ (7)

$$\sum_{i\in I}\sum_{t\in T}x_{it}\leq q\sum_{i\in I}\sum_{t\in T}a_{it}$$ (8)

$$x_{it}\leq a_{it},\;\forall i\in I,\;t\in T$$ (9)

$$x_{it}\geq 0,\;\forall i\in I,\;t\in T$$ (10)

**Table 2:** Parameters and decision variables used in the reserve selection model.

Notation	Description
Parameters:	
*I*	Set of 50x50 km squares (*i = 1*,.* *.* *.,*n*)
*T*	Set of age classes (*t = 1*,.* *.* *.,*m*)
*E*	Set of structural indicators (*e = 1*,.* *.* *.,*o*)
*J*	Set of time periods (*j = 1*,*…*,*q)*
*w*_*e*_	Weight of structural indicator *e*
*p*_*ejit*_	Point of structural indicator *e* in time period *j*, square *i* and age class *t*
*a*_*it*_	Area (ha) of square *i* in age class *t*
*c*_*it*_	Cost ha^-1^ of square *i* and age class *t*
*f*_*ej*_	Discounting function of indicator *e* in time period *j*
*Q*	maximum proportion that can be selected
*B*	available budget (SEK)
Decision variable:	
*x*_*it*_	area (ha) selected in square *i* and age class *t*
Objectives:	
*z*_*e*_	The maximized goal values from phase one
*Y*	The minimized goal value from phase two

In the objective function (Eq 6), the sum of the indicator points in the selected areas is maximized (this sum is referred to as the indicator score). The constraints applied are a budget constraint that limits the total cost (b) of the selected areas (Eq 7), an area constraint that prevents the selected area from exceeding a certain proportion (q) of the total area (Eq 8), a constraint that ensures that the selected area is smaller than the total area (Eq 9) and a non-negativity constraint (Eq 10). Phase one is solved 15 times; once for each indicator when that indicator has weight 1 (*w*_*e*_ = 1) and all other indicators have zero weight. Those 15 goal values are denoted *z*_*e*_.

Phase two [P2] gives a solution that minimizes the sum of the quadratic percentage deviation from each indicator’s goal value and the final value, and is formulated as:

$$\lfloor P2\rfloor \;\text{min}\;y=\sum_{e\in E}{\left(\left(z_{e}-\sum_{j\in J}\sum_{i\in I}\sum_{t\in T}p_{ejit}x_{it}\right)/z_{e}\right)}^{2}$$ (11)

Subject to: Eqs 7–10

This second objective function (11) minimizes the squared difference between the goal and the score for each structural indicator. The deviation is measured as a percentage, so all goals are treated equally. This second problem is convex [41] to guarantee a global optimal solution.

### Selection approaches

We used two selection approaches to evaluate the four discounting functions, one where a budget constrained the selection and one where area constrained the selection. When a budget limited the selection, the area constraint (Eq 8) was omitted, and when area limited the selection the budget constraint (Eq 7) was omitted. We set the budget for selecting reserves at an arbitrary level of 10 billion SEK, which we deemed plausible based on current conservation policy in Sweden, where 6 billion SEK was allocated for establishing reserves during the period 1998–2008 [42]. An area constraint that generated approximately the same indicator score in the first three versions of the discount function was at 5%, (of the total area), hence we chose that level in the area -constrained selections.

## Results

### Development of structural indicators over time

**Fig 1:**
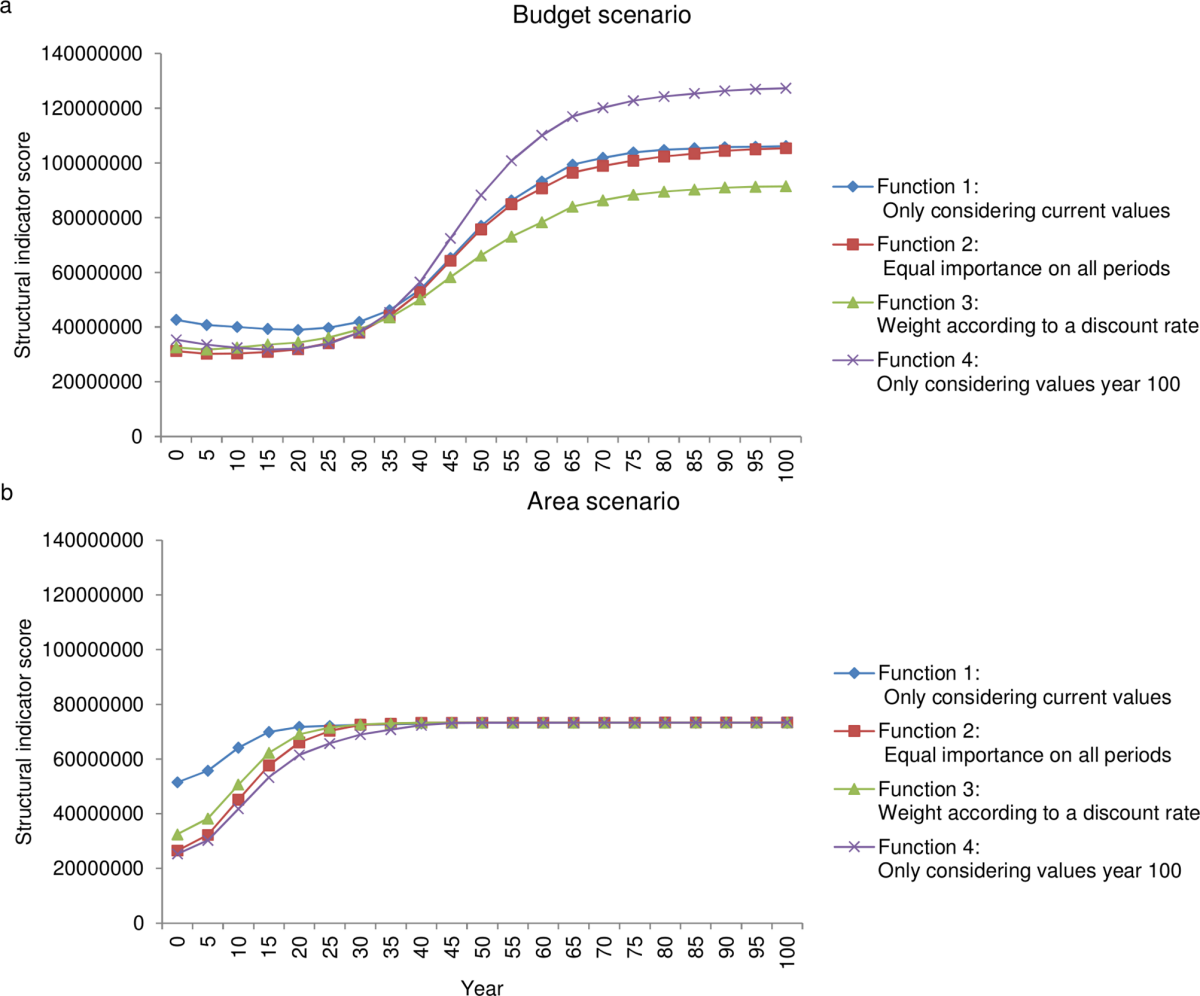
**Changes with time of the structural indicator score per period over 100 years** in (a) the budget-constrained selections and (b) the area-constrained selections for each of the four variants of the discount function, year 0 is the initial state based on NFI data inventoried between 2003 and 2007.

Changes with time in the indicator scores of the selected areas in both the budget-constrained and area-constrained selections were strongly influenced by the discount functions used (Fig 1). Comparisons of indicator scores in the selected areas (based on the simulated points from all age classes and time periods) showed that in the budget-constrained selection the total indicator score over 100 years was highest when only values in year 100 were considered (function 4; see Methods) and lowest when using a discount rate (function 3) (Table 3). In contrast, in the area-constrained selection the total indicator score was highest when only present values were considered (function 1), and lowest when only projected values in year 100 were considered (function 4) (Table 3).

**Table 3:** The total cost (billion SEK), area selected (10 000 ha), and total indicator scores over 100 years (10 million points) of selections yielded by models incorporating the four variants of the discount function for the budget -constrained selections and area-constrained selections.

	Budget-constrained selection	Area-constrained selection
**Discount function 1: current values**		
Cost	10	44
selected area	106	73
total score	154	148
**Discount function 2: all periods**		
Cost	10	43
selected area	105	73
total score	146	140
**Discount function 3: discount rate**		
Cost	10	45
selected area	91	73
total score	131	141
**Discount function 4: only year 100**		
Cost	10	39
selected area	127	73
total score	170	137

The structural indicator score per period was highest for the alternative considering only current values (function 1) for the first 30 years in both budget- and area-constrained selections. For the period >30–100 years the changes in indicator scores yielded by the discounting functions varied. In the budget-constrained selection the indicator score increased most rapidly when considering only values in year 100 (function 4) (Fig 1A), while in the area constrained selection all alternatives generated similar periodic indicator scores (Fig 1B).

**Fig 2:**
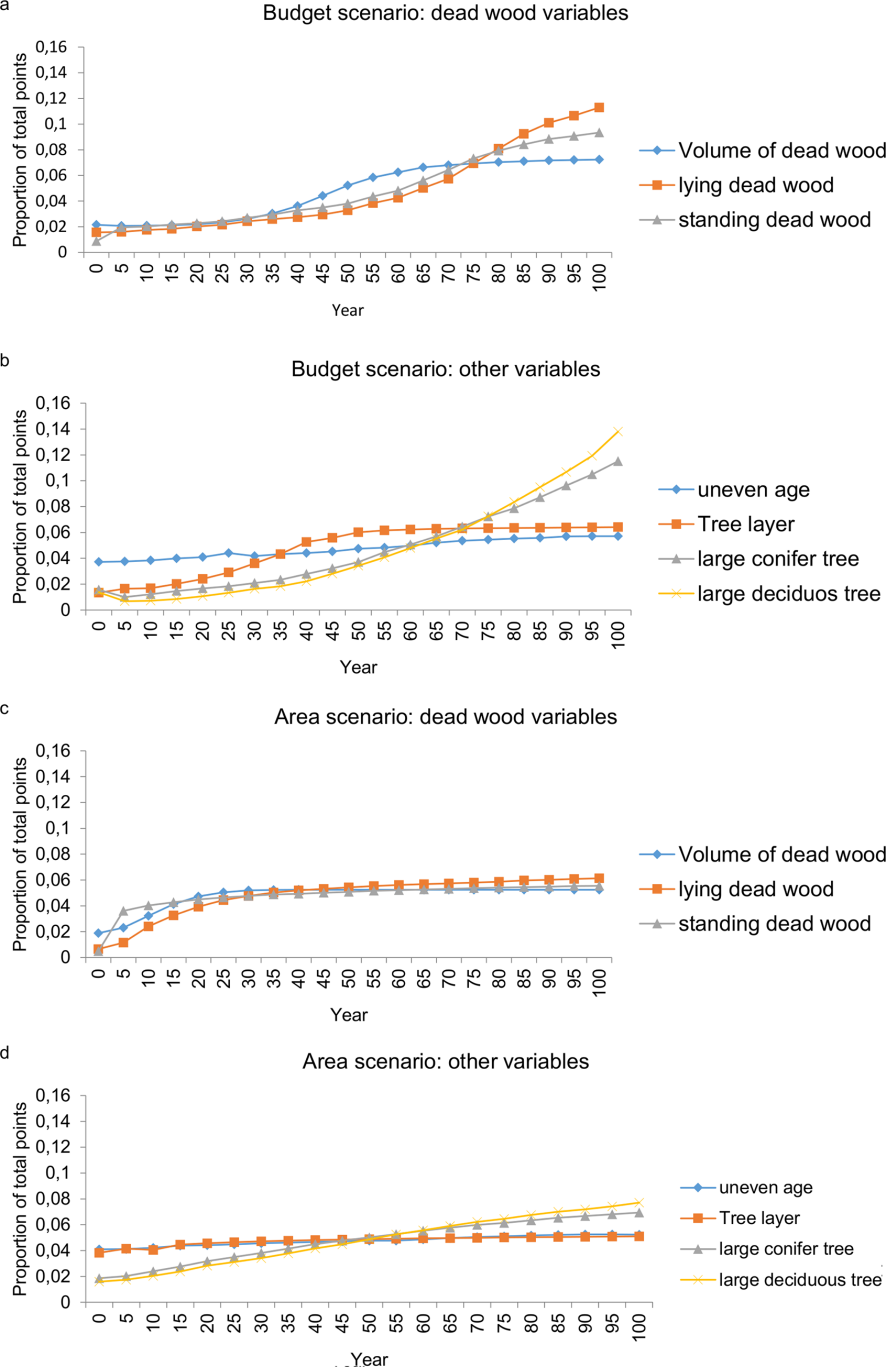
Changes with time of the structural indicators in the selected area over 100 years. Measured as the proportion of the total accumulated points reached in each time step for each indicator or group of indicators: dead wood variables in the (a) budget-constrained selection and (c) area-constrained selection, and other variables in (b) the budget- and (d) area -constrained selections. Year 0 is the initial state based on NFI data inventoried between 2003–2007. Discount function 2 (giving all time periods equal weighting) was applied. To make the figure clearer we grouped the following variables: dead conifer tree standing and dead deciduous tree standing into standing dead wood; dead conifer tree lying and dead deciduous tree lying into lying dead wood; large pine and large spruce into large conifer tree; and large birch, large aspen and large deciduous tree (other than birch or aspen) into large deciduous tree.

Analysis of the development of the individual indicators when giving equal importance to all time periods (function 2) showed that points increased more rapidly for the budget-constrained selection than for the area-constrained selection. For the budget-constrained selection points for lying dead wood, standing dead wood and large trees increased rapidly while those for total volume dead wood, tree layer and uneven age did not change much over time (Fig 2A–2D).

### Age distribution

**Fig 3:**
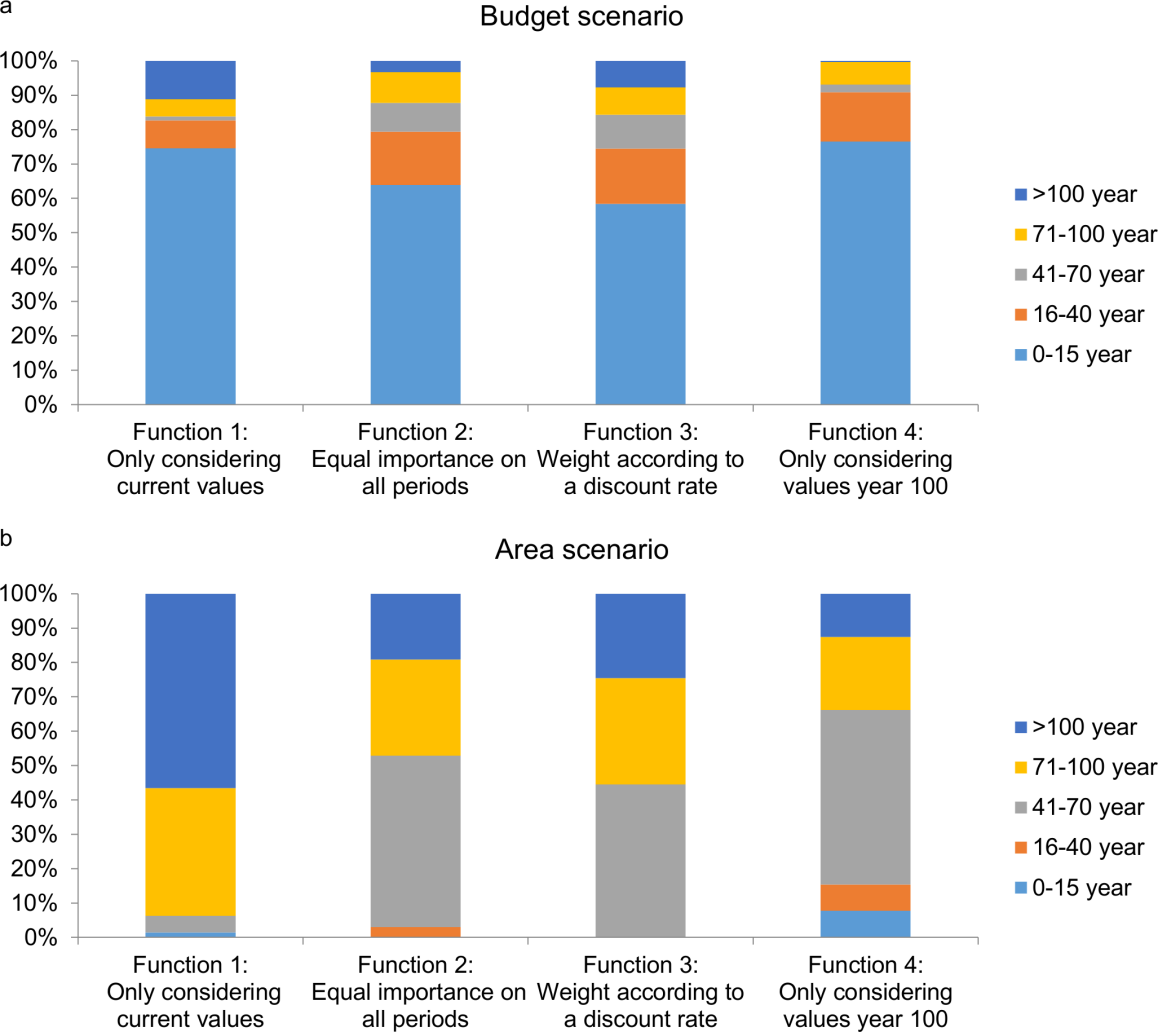
**Optimal age distribution of reserves in boreal Sweden** obtained by applying the four variants of the discount function in (a) the budget-constrained selections and (b) the area-constrained selections.

The age distribution of the selected forest varied depending on the discounting function, and differed strongly between budget-constrained and area-constrained selections. In the budget-constrained selection, the age class 0–15 years dominated in all cases with a proportion between 76% (function 4) and 60% (function 3) (Fig 3A). Proportions of the three oldest age classes (41–70, 71–100 and >100 years) selected were low under all of the discounting functions in the budget-constrained selection, ranging from 0.3% to 11% (Fig 3A).

In contrast, in the area-constrained selection the proportions of age class 0–15 years were consistently low and at most 8%, when only the values for year 100 (function 4) were considered (Fig 3B). The oldest age class was more prominent in this selection approach, and highest when only current values were considered (function 1) (57%), but considerably lower in the other alternatives, ranging from 13% (function 4), to 25% (function 3) (Fig 3B). For the area-constrained selections forests >40–100 years old dominated the selections for discount functions 2,3,4 while for discount function 1 forests >70 years old comprised 95% of the area.

### Budget-constrained versus area-constrained selections

Outputs of the area-constrained models were approximately four times more costly and selected only 70% of the area compared with the budget-constrained models (Table 3). Discount function 4 in the budget-constrained models yielded the largest area and the highest total score of all selections. For the area-constrained models, there were small differences between functions. The variations in total score between functions were smaller for the area-constrained models than the budget-constrained models (Table 3).

## Discussion

### Considering future states is important

Overall, our study shows that the most efficient selection of reserves strongly depends on whether current or future structural diversity is considered. This was especially true for the budget-constrained models in which the most efficient solution, in the sense of a high accumulated structural score and a large selected area, was when only year 100 was considered (discount function 4). According to the applied models the structural diversity of young and middle-aged forests can clearly increase substantially in a long-term perspective, and thus the 100 year target was important to consider. Discount functions also affected forest-age distributions with the proportion forest <15 years old varying between 60% and 76% for the budget-constrained selections, and between 0% and 8% for the area-constrained selections.

### Hypotheses

Our first hypothesis, that the total scores over 100 years would be higher if larger weighting was applied to future values than current values in the selection, was only partly supported. In the budget-constrained selection, this was true for the alternative only considering the value in year 100 (function 4) but not for functions 2 and 3. Further, our results were not consistent with our second hypothesis, that young forests would be selected to an even higher degree if future values were stressed in the budget-constrained selection alternatives. Instead, the proportion of young forest was lower in the two alternatives considering all time periods (functions 2 and 3). However, we found a marginal increase when only the value in year 100 (function 4) was considered. This could be attributed to the relatively rapid increases in most structural indicators in the middle aged forests, which today are highly impoverished due to previous management practices.

### Structures and species

We only analysed selections based on structural variables due to the large body of scientific evidence pointing to a strong connection between high structural diversity and rich species diversity [1], and also that high-quality structural data are available through the Swedish NFI. However, forest history and connectivity also influence species’ occurrences [43], and the probability of finding associated species at a site with high structural diversity will vary over time and space. If a forest landscape has a history of low structural diversity and a site is very isolated, species diversity will probably be relatively low. An interesting extension of our study would therefore be to repeat the analyses with data on species to capture how the history and spatial location of possible habitats affect species occurrences over time and space [44]. Extensive databases on species occurrences in Sweden are available in citizen-science based databases that could be used in such analyses [45]

### Young forests in reserve networks

The solutions when the target was to limit the cost included a considerably larger total area than when the target was to only reach a certain area limit. In the budget-constrained models a much higher proportion of young forest was collected than in area-constrained models where old forests prevailed. This is because young forests are much cheaper than old forest, due to their low tree volumes (the net present value per ha for forest >100 years old was approximately three times higher than for forest <15 years old). Higher structural richness could be reached at a quarter of the cost and over almost twice the area in the budget-constrained model than in the area-constrained model when the discount function only considered the values in 100 years (function 4) Thus, the cost-efficient solutions could apparently provide both major current savings for taxpayers and a much more extensive reserve network with high structural qualities in the future. Nevertheless, caution must be raised against a drastic reorientation of the current forest strategy, i.e., from prioritizing protection of old forest to setting aside young age stages. In regions with long histories of industrial forestry, like northern Europe, landscapes have become highly fragmented with few remnant old forests, and relatively intact landscapes that are still present need to be prioritized due to their unique composition. However, in regions where there is a lack of high-quality old forests, younger forests may provide important alternatives for reservation.

The choice of time-span is also crucial, and can affect judgments of possible options, especially in a dynamic and changing world, but there is no simple scientific guide for choosing the optimal time horizon [46]. Mönkkönen et al. [47] evaluated alternative strategies for conservation in managed boreal forest landscapes over time and found that their relative effectiveness depended on the time-frame. We chose 100 years because this is a normal rotation time in a managed boreal forest in northern Europe, and also structural features like dead wood and large-diameter trees develop substantially during this time span [34,48,49] Still, for some structures, like old trees, considerably longer time periods would be needed since the life span may exceed several hundred years for most tree species. Nevertheless, a shorter time horizon may be more appealing for decision-makers, since it is easier to relate to a closer, future end point. However, we also found variations in relative effectiveness with time. If we had chosen a shorter time-period, e.g. 30 years, alternatives that consider future values would have been disfavored since the discount function including only present values generates the highest total score per period in the first 30 years in both the budget- and area-constrained models.

### Incorporating future risks

We used storm as an example of future risk, since a storm would reduce the projected future values of the indicators linked to living trees. We also assumed that this risk would increase with time since the frequency and intensity of storms are expected to increase due to climate change [50,51]. However, the relationships between storms and our indicators are not simple, since storms would lead to raised levels of dead wood, a substrate important to many species. Thus, attempts should be made to account for both positive and negative effects of risk factors in future analyses. Climate change is expected to have major effects on all levels of biodiversity, but there are large variations in projected responses [52]. Despite those uncertainties, climate change should still be considered in conservation planning [53], for example by applying a systematic replacement of protected areas over time to adjust for changes in the environment [23]. Conservation planners need to adapt and search for alternative approaches to be able to cope with the large uncertainties ahead, and reflect our desire to protect biodiversity for today or for future generations.

### Practical implications

Reserves in boreal northern Europe could be selected more rationally. A key possibility addressed here that has been largely neglected to date [22,46,47], is that the effectiveness of selections could potentially be enhanced by considering future values. We have found clear indications that optimal strategies differ depending on the applied discounting functions and time-frames. In Swedish boreal forests considered here, this strongly affects the age-distribution of the selected areas, particularly favoring young and middle-aged forests when timeframes are long. Future studies should develop planning approaches that can consider future development of the selected areas and refine the measure of structural indicators, which in turn can be used as a foundation for conservation planners to formulate more effective conservation decisions.

## Supporting Information

S1 Dataset Supporting data. (XLSX)
